# Early reduction of skin potassium without sodium accumulation in the pathogenesis of salt sensitivity in primary aldosteronism

**DOI:** 10.3389/fphar.2025.1575972

**Published:** 2025-04-29

**Authors:** Petr Mlejnek, František Liška, Jan Šilhavý, Kateřina Večerková, Miroslava Šimáková, Michal Pravenec, Theodore W. Kurtz

**Affiliations:** ^1^ Institute of Physiology, Czech Academy of Sciences, Prague, Czechia; ^2^ Institute of Biology and Medical Genetics, First Faculty of Medicine, Charles University and General University Hospital, Prague, Czechia; ^3^ Laboratory of Genomics and Bioinformatics, Institute of Molecular Genetics, Czech Academy of Sciences, Prague, Czechia; ^4^ Department of Informatics and Chemistry, University of Chemistry and Technology, Prague, Czechia; ^5^ Department of Laboratory Medicine, University of California, San Francisco, San Francisco, CA, United States

**Keywords:** hyperaldosteronism, aldosterone, skin, hypokalemia, salt sensitivity, hypertension, sodium, potassium

## Abstract

**Introduction:**

Primary aldosteronism is the most common form of secondary hypertension and blood pressure salt sensitivity. In the setting of hyperaldosteronism and a high-salt diet, disturbances in tissue sodium and potassium levels may contribute to salt sensitivity. This study aimed to determine whether aldosterone-dependent changes in tissue and plasma sodium and potassium concentrations occur before or after the development of salt sensitivity and hypertension in a rat model of primary aldosteronism. Previous studies in this model show that aldosterone-dependent salt sensitivity develops after 7–10 days on a high-salt diet. A secondary objective was to investigate differences in skin gene expression between aldosterone-treated rats and vehicle-treated controls.

**Methods:**

Unilaterally nephrectomized male Sprague-Dawley rats received continuous infusions of aldosterone or vehicle while being fed a high-salt diet. Electrolyte concentrations in plasma, carcass, and skin were measured after 2 and 14 days of high-salt feeding. Tissue sodium and potassium concentrations were determined by atomic absorption spectroscopy and expressed as mmol/g tissue dry weight, while plasma ions (mmol/L) were measured using ion-selective electrodes. RNA sequencing (RNAseq) was used to identify differentially expressed genes in the skin, and gene set enrichment analysis (GSEA) was performed to explore biological processes associated with aldosterone treatment.

**Results:**

After 2 days on the high-salt diet, aldosterone-treated rats showed significantly lower skin and plasma potassium concentrations compared to vehicle-treated controls, while sodium concentrations in the carcass, skin, and plasma did not differ significantly. At 14 days, aldosterone-treated rats continued to exhibit lower plasma potassium levels, although skin potassium differences were no longer significant. Carcass sodium concentrations were significantly higher in aldosterone-treated rats at 14 days. GSEA revealed that, at 2 days, aldosterone treatment affected biological processes related to electrolyte homeostasis and hyperosmotic responses. At 14 days, biological processes related to muscle function and calcium ion transport were significantly altered.

**Conclusion:**

Aldosterone-treated rats on a high-salt diet for 2 days had lower skin and plasma potassium levels compared to salt-loaded controls, suggesting early potassium depletion precedes significant sodium accumulation and blood pressure increases. These findings raise the possibility that early potassium depletion contributes to the development of aldosterone-induced salt sensitivity. Further studies with detailed time-course analysis will be of interest to elucidate the role of early potassium depletion in increasing vascular resistance and triggering aldosterone-dependent salt sensitivity and hypertension.

## 1 Introduction

Primary aldosteronism is a common disorder involved in the development of blood pressure salt sensitivity and salt-dependent hypertension (Funder, 2015; Funder, 2022). In animal models, increased plasma aldosterone – achieved by genetic or pharmacologic methods – greatly enhances salt sensitivity and the hypertensive effects of high-salt diets (Gu et al., 2017; Blasi et al., 2003). In contrast, elevating plasma aldosterone levels in the context of very low salt intake has little or no effect on blood pressure (Shibata et al., 2011). Recent studies in a rat model of primary aldosteronism demonstrated that aldosterone enhances salt sensitivity and augments salt-induced hypertension by amplifying salt-dependent increases in systemic vascular resistance while reducing cardiac output (Kurtz et al., 2023).

It has been proposed that electrolyte disturbances in tissues such as skin, brain, and vasculature may promote increased vascular resistance and contribute to salt-sensitivity and hypertension (Rossitto and Delles, 2022; Oberleithner et al., 2007; Oberleithner et al., 2009; Kusche-Vihrog et al., 2014; Helle et al., 2013). Forty years ago, Williams and colleagues reported that patients with primary aldosteronism exhibited elevated total body sodium, and reduced total body potassium (Williams et al., 1984). Recently, Torresan and colleagues observed that surgical cure of unilateral primary aldosteronism in humans was associated with significant increases in skin and plasma potassium concentrations without changes in sodium levels (Torresan et al., 2024). Although it is not possible to measure tissue electrolytes in clinical practice, detection of hypokalemia in plasma samples can sometimes be a clue to the presence of hyperaldosteronism. However, there is little information on the time course of onset of plasma and tissue electrolyte disturbances during the pathogenesis of hypertension induced by high salt intake in hyperaldosteronism. If aldosterone-dependent disturbances in tissue or plasma electrolyte concentrations precede salt-induced increases in blood pressure, they might be a cause of the hypertension. If the aldosterone-dependent electrolyte disturbances occur only after the onset of increased blood pressure, they might simply be a consequence of the hypertension. In the current study, in a rat model of primary aldosteronism, we investigated skin and plasma electrolyte concentrations after 2 days and 14 days of administering a high salt diet. As shown in previous studies in this model, these represent time points occurring before and after the development of aldosterone-dependent salt sensitivity and hypertension (Kurtz et al., 2023).

## 2 Methods

### 2.1 Animals

We used 10-week-old male Sprague-Dawley rats from Charles River Germany. Rats were housed separately and initially given unrestricted access to tap water and a pelleted purified AIN-76A diet containing 0.26% NaCl and 0.36% potassium (diet No.100000 from Dyets, Inc., Bethelem, PA). This diet provides approximately twice the minimum amount of salt recommended to support growth and reproduction in rats (National Research Council Board on Agriculture Committee on Animal Nutrition Subcommittee on Laboratory Animal Nutrition, 1995). However, it is lower than the typical amount of salt present in ordinary rat chow which ranges from ∼0.5% to ∼1.2% NaCl (Martus et al., 2005; Gohar et al., 2022). We refer to this 0.26% salt diet as the “low salt” diet. This low salt diet was provided until the animals were ready for the studies that were conducted on the high salt (4% NaCl diet) as described further below. All animal experiments were conducted in compliance with the Animal Protection Law of the Czech Republic and were approved by the Ethics Committee of the Institute of Physiology, Czech Academy of Sciences, Prague, protocol number 15–2022-P.

### 2.2 Experimental protocol

The experimental time line is shown in Supplementary Figure S1. Rats weighing approximately 300 g underwent unilateral nephrectomy as previously described (Kurtz et al., 2023). After surgery, all rats were individually housed in their cages and provided free access to tap water and the low salt diet. Four weeks after nephrectomy, animals were randomized to receive a continuous subcutaneous infusion of either aldosterone or vehicle via subcutaneously implanted osmotic minipumps (2 pumps/rat) as previously described (Kurtz et al., 2023). The aldosterone was continuously infused at a dose of 1.5 μg/h with a volume flow rate of 0.3 μL/h. This dose of aldosterone has been reported to increase serum aldosterone levels to approximately 2000 pg/mL in Sprague Dawley rats (Reil et al., 2012). This is a 4-fold increase over the serum level of aldosterone observed in Sprague Dawley rats ingesting normal rat chow containing 0.9% NaCl (Reil et al., 2012).

After implantation of the osmotic minipumps, each rat was returned to its home cage and maintained on the low salt (0.26% NaCl) diet and tap water and allowed to recover from minipump implantation for 2 weeks. After the 2 weeks recovery, all rats were started on the high salt (4% NaCl) diet and tap water *ad libitum*. The high salt diet contained 4% NaCl (AIN-76A diet with 4% NaCl, diet No. 113756 from Dyets Inc., Bethlehem, PA) and was identical in composition to the low salt diet except with respect to salt content. This protocol is identical to the protocol we previously used to study the time course of changes in blood pressure, cardiac output, and vascular resistance induced by high salt diet in rats with hyperaldosteronism (Kurtz et al., 2023). Rats were killed by cervical dislocation. Carcasses, skin and plasma for electrolyte measurements were collected from aldosterone and vehicle treated rats after 2 days on high salt diet (N = 5 per group) and 14 days on high salt diet (N = 5 per group).

### 2.3 Tissue and plasma electrolyte measurements

Intestines and stomach were completely removed from rats to exclude remains of chow. The skins and carcasses (body without skin, intestines and stomach) were weighed to determined wet weight (WW). Biopsy samples of interscapular skin (1 cm × 1 cm) were used for RNAseq analysis. The carcasses and skin were then desiccated at 105°C for 36 h to determine dry weight (DW). The difference between WW and DW is considered as tissue water content. Ashing of carcasses was performed at 200°C and 450°C for 20 h at each temperature level, and the bones were sieved from the carcass ashes. Ashing of skins was performed at 200°C and 450°C for 10 h at each temperature level. Ashes were sent to ALS Czech Republic, Ltd. (Prague, Czech Republic) for analysis of Na^+^ and K^+^ concentrations by atomic absorption spectrometry. Chloride concentrations in the ashes were measured by titration with 0.1N silver nitrate. Plasma Na^+^, K^+^ and Cl^−^ concentrations were measured by the State Veterinary Institute (Prague, Czech Republic) using ion selective electrodes. All chemical parameters in tissues are expressed as relative values corrected to dry weights of carcass and skin. In the present manuscript, we refer to the tissue electrolyte values corrected to dry weight as tissue electrolyte “concentrations.” Plasma electrolyte concentrations are expressed as mmol/L.

### 2.4 Gene expression analysis

We used the RNAseq method for determination of gene expression levels in the skin biopsies as previously described (Šilhavý et al., 2022). Briefly, skin biopsy samples were flash frozen using liquid nitrogen and stored at −80°C. The frozen samples were powdered by mortar and pestle in liquid nitrogen and homogenized using TissueLyser (Qiagen) for 10 min at 50 Hz in the lysis buffer RLT Plus (Qiagen) with addition of 1% β-mercaptoethanol. Total RNA was isolated using RNeasy Plus Mini Kit (Qiagen) according to the manufacturer’s instructions with proteinase K treatment of the lysates and on-column DNAse I treatment. RNA was quantified using UV spectrophotometry and 2,100 Bioanalyzer and the RNA-6000 Nano-LabChip (Agilent) was used to assess RNA integrity. Only samples showing RNA integrity numbers (RIN) above eight were used for further analysis. The extracted RNA was used to prepare cDNA libraries for sequencing on the Illumina NextSeq^®^ 500 instrument using 76bp single-end configuration. Read quality was assessed by FastQC. We used the bioinformatic pipeline nf-core/rnaseq version 3.12.0 for read processing. Individual steps included removing sequencing adaptors with Trim Galore, mapping to reference genome mRatBN7.2 (Ensembl annotation version 111) with STAR (Dobin et al., 2013), and quantifying expression on gene level with Salmon (Patro et al., 2017). Per gene mapped counts served as input for differential expression analysis using DESeq2 R Bioconductor package. Prior to the analysis, genes not expressed by ten transcripts in at least eight samples were discarded. Gene set enrichment analysis (GSEA) was performed with the algorithm in the clusterProfiler R Bioconductor package to identify Gene Ontology (GO) Biological Processes with normalized expression scores significantly affected by aldosterone-treatment compared with vehicle-treatment.

### 2.5 Statistical analysis

The primary outcome variables were tissue concentrations (carcass tissue and skin tissue) and plasma concentrations of sodium and potassium. Data are expressed as means and 95% confidence intervals. We used the estimation statistics and graphics routines on the web application www.estimatestats.com to test for and display differences in group means (Ho et al., 2019; Claridge-Chang and Assam, 2016). For analyzing differences in group means, we also include P values that were determined by permutation testing to indicate the probability of observing the effect size (or greater), assuming the null hypothesis of zero difference is true. For each permutation P value, at least 5,000 reshuffles of the control and aldosterone data were performed. P values <0.05 were considered significant. Correction for multiple testing with the Benjamini–Hochberg procedure was used to control the false discovery rate (FDR) in the gene expression analysis and gene set enrichment analysis. Specifically, differentially expressed genes were defined by setting the FDR <0.05 for genes with a log fold change of ≥1 (absolute value). In the GSEA, normalized enrichment scores with FDR of <0.001 were considered statistically significant. Normalized expression scores >1.0 or < −1.0 were considered to indicate potential enrichment and scores >2.0 or < −2.0 were considered to indicate strong enrichment.

## 3 Results

### 3.1 Tissue and plasma electrolyte concentrations and water content

#### 3.1.1 At 2 days on the high salt diet

**FIGURE 1:**
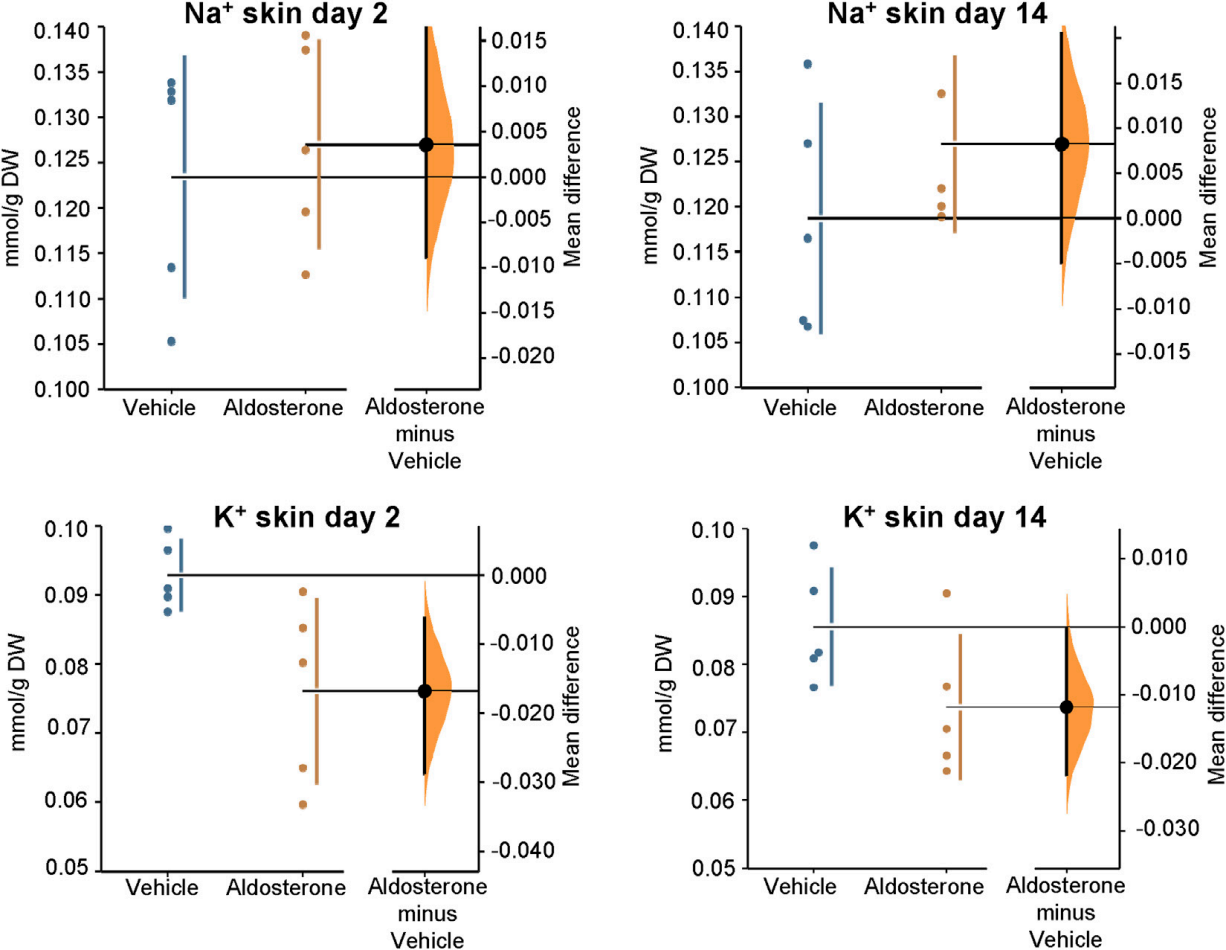
Sodium and potassium concentrations in the skin. Within 2 days of initiating the high-salt diet, aldosterone-treated rats exhibited significantly lower skin potassium concentrations than vehicle-treated controls (P = 0.016). At 14 days of the high salt diet, skin potassium appeared lower in the aldosterone group than in controls but the difference did not reach statistical significance (P = 0.073). Skin sodium concentrations were not significantly different between the two groups at 2 days or 14 days of the high salt diet. The Gardner–Altman estimation plot (Ho et al., 2019) displays the mean difference in electrolyte concentrations between the aldosterone and vehicle-treated rats. The left axis shows the individual group data, while the right floating axis displays the bootstrap sampling distribution of the mean group difference, represented as a dot with vertical error bars indicating the 95% confidence interval. DW, dry weight.

**FIGURE 2 F2:**
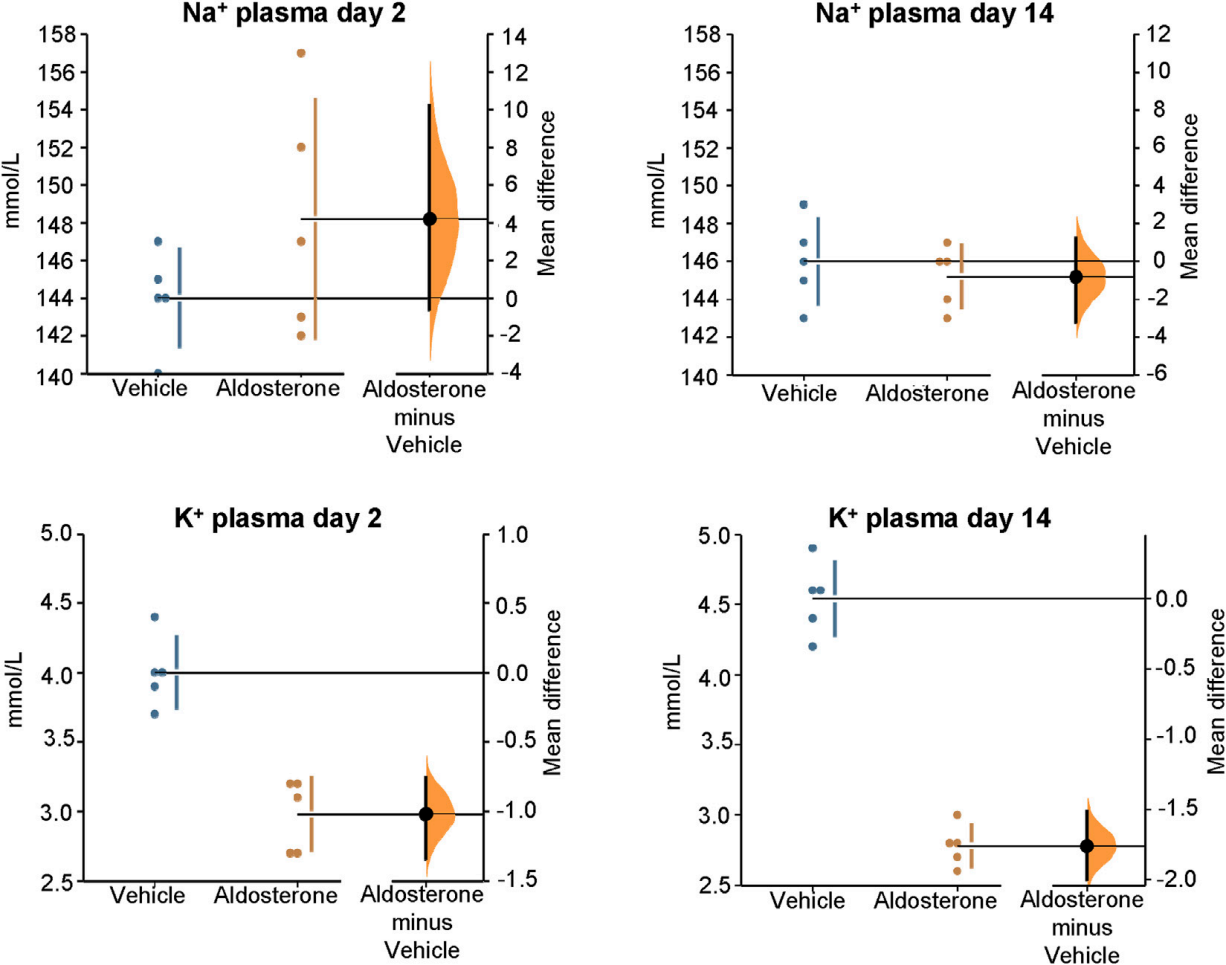
Sodium and potassium concentrations in plasma. Within 2 days of initiating the high-salt diet, aldosterone-treated rats had significantly lower plasma potassium concentrations compared with vehicle-treated controls (P = 0.008). After 14 days on the high-salt diet, plasma potassium concentrations remained significantly reduced in the aldosterone-treated rats P = 0.0076). Plasma sodium concentrations were not significantly different between groups on day 2 or day 14. The Gardner–Altman estimation plot (Ho et al., 2019) shows the mean difference in electrolyte concentrations between the two groups. Data for both groups are plotted on the left axis, and the right floating axis presents the bootstrap sampling distribution of the mean group difference, depicted as a dot with vertical error bars representing the 95% confidence interval.

**FIGURE 3:**
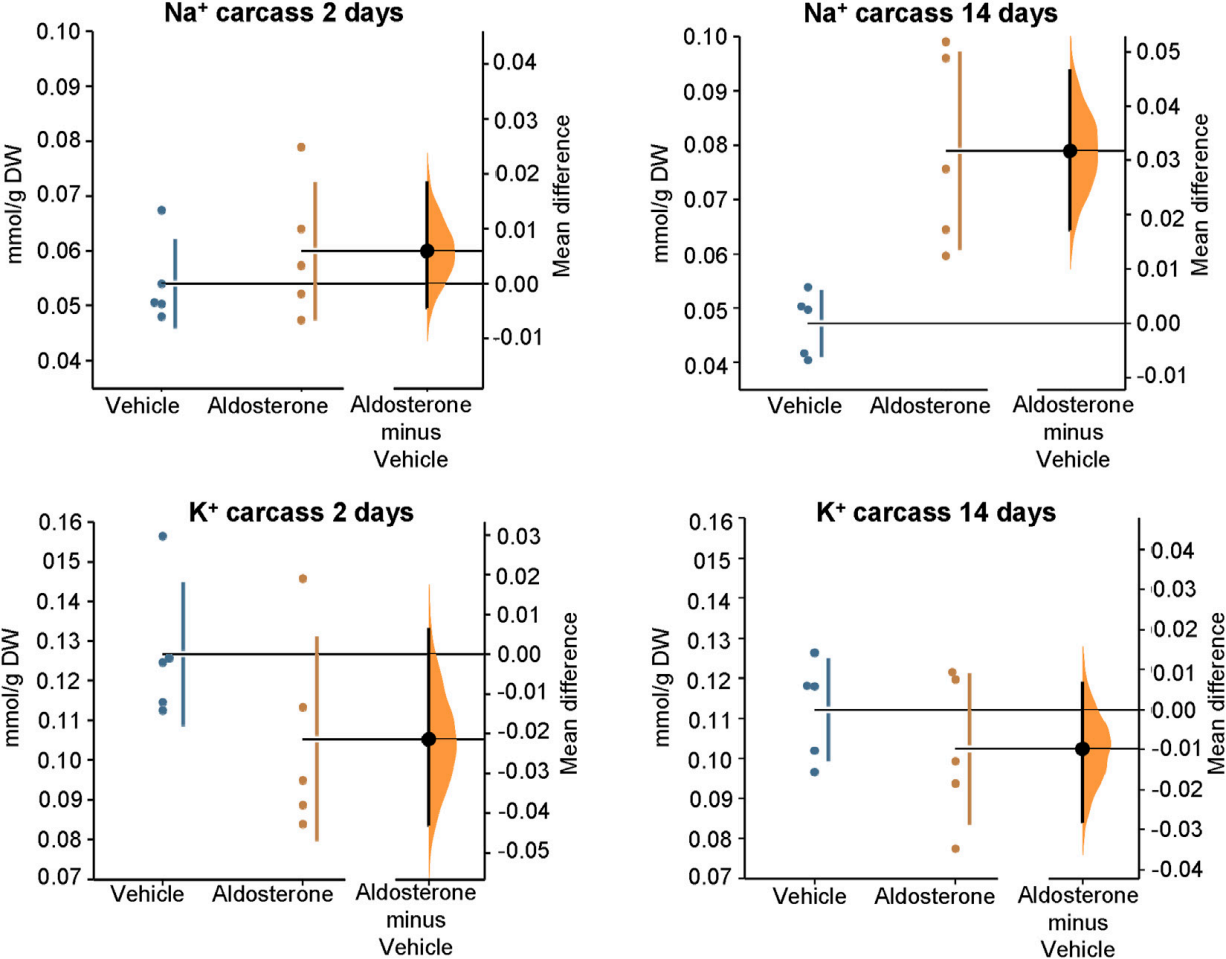
Sodium and potassium concentrations in the carcass. Within 2 days of initiating the high-salt diet, aldosterone-treated rats showed a trend toward lower carcass potassium concentrations and greater sodium concentrations compared with vehicle-treated controls; however, the group differences were not statistically significant. After 14 days on the high-salt diet, carcass sodium concentrations were significantly greater in aldosterone-treated rats compared with vehicle-treated controls (P = 0.0079). Group differences in carcass potassium concentrations were not significantly different after 14 days on the high salt diet. The Gardner–Altman estimation plot (Ho et al., 2019) illustrates the mean difference in electrolyte concentrations between the two groups. The left axis displays the data for both groups, while the right floating axis shows the bootstrap sampling distribution of the mean group difference, depicted as a dot with vertical error bars representing the 95% confidence interval. DW, dry weight.

In rats fed the high salt diet for 2 days, aldosterone-treated rats had significantly lower potassium concentrations in both skin and plasma compared with vehicle-treated controls (Figures 1,2 [Fig F2]; Table 1). In contrast, sodium concentrations in skin, plasma, and carcass did not differ significantly between groups (Figures 1–3; Table 1).

**TABLE 1:** Means and 95% CI of variables in rats during treatment with aldosterone or vehicle.

Traits	Vehicle + salt for 2 days	Aldosterone + salt for 2 days	Vehicle + salt for 14 days	Aldosterone + salt for 14 days
Body weight, g	414 (394–434)	410 (398–422)	439 (408–470)	416 (409–423)
Carcass weight, g)	293 (276–310)	297 (285–310)	308 (282–334)	303 (295–311)
Carcass dry weight (g)	91 (87–95)	94 (88–100)	99 (88–110)	94 (91–97)
Carcass water (mL)	202 (189–215)	204 (197–211)	209 (194–224)	209 (204–214)
Carcass relative water (mg/g WW)	0.685 (0.667–0.693)	0.689 (0.678–0.700)	0.679 (0.662–0.696)	0.679 (0.673–0.685)
Skin weight (g)	74 (65–83)	74 (61–87)	82 (73–91)	66 (50–82)
Skin dry weight (g)	31 (28–34)	31 (24–38)	36 (34–38)	29 (21–37)
Skin water (mL)	43 (37–49)	42 (36–48)	46 (39–53)	37 (28–46)
Skin relative water (mg/g WW)	0.577 (0.552–0.602)	0.581 (0.570–0.592)	0.560 (0.527–0.593)	0.560 (0.541–0.579)
Carcass Na^+^ (mmol/g DW)	0.054 (0.044–0.064)	0.06 (0.045–0.075)	0.047 (0.04–0.054)	0.079 (0.057–0.101)^c^
Carcass K^+^ (mmol/g DW)	0.127 (0.105–0.148)	0.105 (0.0374–0.136)	0.112 (0.097–0.128)	0.102 (0.079–0.125)
Carcass Cl^−^ (mmol/g DW)	0.017 (0.014–0.021)	0.017 (0.015–0.019)	0.015 (0.011–0.0196)	0.012 (0.004–0.020)
Skin Na^+^ (mmol/g DW)	0.123 (0.107–0.139)	0.127 (0.114–0.142)	0.119 (0.103–0.134)	0.127 (0.115–0.139)
Skin K^+^ (mmol/g DW)	0.093 (0.087–0.099)	0.076 (0.059–0.092)^a^	0.086 (0.075–0.096)	0.074 (0.061–0.087)^f^
Skin Cl^−^ (mmol/g DW)	0.046 (0.023–0.068)	0.051 (0.025–0.076)	0.044 (0.038–0.0499)	0.041 (0.021–0.061)
Plasma Na^+^ (mmol/L)	144 (141–147)	148 (140–156)	146 (143–149)	145 (143–147)
Plasma K^+^ (mmol/L)	4.0 (3.7–4.3)	2.8 (2.5–3.1)^b^	4.5 (4.2–4.8)	2.8 (2.77–2.83)^d^
Plasma Cl^−^ (mmol/L)	104 (101–107)	104 (96–112)	106 (104–108)	101 (98–104)^e^

CI, confidence intervals. Sample size is n = 5 per group. P values <0.05 in two-tailed permutation testing for differences in means between aldosterone and vehicle groups fed the 4% salt diet for same amount of time denoted by:

^a^ P = 0.016.

^b^ P = 0.008.

^c^ P = 0.0079.

^d^ P = 0.0076.

^e^ P = 0.008.

^f^ P = 0.073.

#### 3.1.2 At 14 days on the high salt diet

In rats fed the high salt diet for 14 days, plasma potassium remained significantly lower in aldosterone-treated rats relative to controls (Figure 2; Table 1). Although mean skin potassium concentrations were lower in the aldosterone group *versus* controls, the difference did not reach statistical significance (P = 0.073), nor did carcass potassium levels (Figures 1, 3; Table 1).

Conversely, carcass sodium concentrations were significantly higher in the aldosterone-treated rats compared with controls (Figure 3; Table 1), while sodium concentrations in skin and plasma remained similar between groups (Figures 1,2 [Fig F2]). Plasma chloride concentrations were significantly reduced in aldosterone-treated rats after 14 days (Table 1). No significant group differences in water content were observed in the skin or carcass at either time point (Table 1).

### 3.2 Gene expression profiles in the skin

#### 3.2.1 At 2 days on the high salt diet

To search for biologic processes in skin affected by aldosterone treatment, we performed gene set enrichment analysis (GSEA) of gene expression results in skin biopsies from aldosterone-treated rats relative to vehicle-treated rats. In rats fed the high salt diet for 2 days, GSEA identified a total of six biological processes where the enrichment scores were significantly affected in the comparison of aldosterone treated rats relative to the control rats. All six of these biological processes are listed in Table 2. Among these processes, three were related to anion homeostasis and one to hyperosmotic responses. The negative normalized enrichment scores (approximately −1.9) indicate that most genes in these sets were downregulated in the aldosterone group compared with controls. The expression levels of all individual genes after 2 days of the high salt diet are posted on ArrayExpress at https://www.ebi.ac.uk/biostudies/arrayexpress website. Deposition number E-MTAB-14749.

**TABLE 2:** Selected biological processes in skin affected by aldosterone treatment in rats as revealed by gene set enrichment analysis.

Biological processes significantly affected by aldosterone	Enrichment score	Enrichment score normalized	P-value	FDR
After 2 days on high salt diet				
Cellular anion homeostasis	−0.704	−1.94	0.000826	0.000926
Monovalent inorganic anion homeostasis	−0.687	−1.92	0.000709	0.000926
Chloride ion homeostasis	−0.687	−1.92	0.000709	0.000926
Hyperosmotic response	−0.620	−1.91	0.000726	0.000926
Axo-dendritic transport	−0.472	−1.72	0.000921	0.000926
Negative regulation of cell division	−0.755	−1.94	0.000926	0.000926
After 14 days on high salt diet				
Myofibril assembly	0.857	2.58	1.00e-10	8.28e-10
Striated muscle cell development	0.853	2.57	1.00e-10	8.28e-10
Sarcomere organization	0.87	2.50	1.00e-10	8.28e-10
Striated muscle contraction	0.736	2.49	1.00e-10	8.28e-10
Skeletal muscle contraction	0.874	2.48	1.00e-10	8.28e-10
Muscle cell development	0.705	2.44	1.00e-10	8.28e-10
Muscle contraction	0.687	2.44	1.00e-10	8.28e-10
Multicellular organismal movement	0.815	2.41	1.00e-10	8.28e-10
Musculoskeletal movement	0.815	2.41	1.00e-10	8.28e-10
Energy derivation by oxidation of organic compounds	0.678	2.41	1.00e-10	8.28e-10
Regulation of calcium ion transmembrane transport	0.6	2.04	5.44E-10	3.81E-09
Regulation of calcium ion release into the cystosol by SR	0.813	2.08	2.34E-6	6.01E-06
Regulation of calcium ion transmembrane transporter activity	0.674	2.10	2.03E-08	8.86E-08

FDR, false discovery rate; SR, sarcoplasmic reticulum.

#### 3.2.2 After 14 days of the high salt diet

Salt loading for 14 days was associated with far more changes in gene expression than salt loading for 2 days. In rats fed the high salt diet for 14 days, aldosterone treatment was associated with significant effects on genes involved in 687 biological processes (see excel Supplementary Table S1 showing all 687 affected processes). Of these 687 biological processes, the top 10 processes with the highest positive normalized enrichment scores (all with false discovery rates of 8.28e–10) are displayed in Table 2. These processes primarily involved muscle function (e.g., muscle contraction) and had normalized enrichment scores greater than 2.4, indicating strong upregulation (Table 2). We also identified three processes related to calcium ion transport and to release of calcium into the cytosol with enrichment scores greater than 2.0, indicating strong upregulation in the aldosterone-treated group relative to controls (Table 2). Some of the specific genes involved in these biological processes and that were upregulated in aldosterone-treated animals are discussed further below. The expression levels of all individual genes after 14 days on the high salt diet are posted on ArrayExpress at https://www.ebi.ac.uk/biostudies/arrayexpress website. Deposition number E-MTAB-14749.

## 4 Discussion

### 4.1 Skin potassium

Recent attention has focused on the role of the skin in salt sensitivity and hypertension (Helle et al., 2013; Johnson et al., 2016; Chachaj et al., 2023). Much of the literature has emphasized sodium accumulation in the skin and its potential effects on vascular resistance via immunomodulatory and inflammatory pathways (Rossitto and Delles, 2022; Miyauchi et al., 2024). In contrast, recent findings by Torresan et al. (2024) suggest that skin potassium depletion may play a critical role in the pathogenesis of increased blood pressure in primary aldosteronism. They observed that, following surgical cure of unilateral primary aldosteronism, skin and plasma potassium concentrations increased without changes in sodium levels. In our study, aldosterone-treated rats fed a high-salt diet for 2 days exhibited substantial hypokalemia and reduced skin potassium concentrations compared with vehicle-treated controls, while sodium concentrations in skin, carcass, and plasma were unchanged. These findings are consistent with the results of Torresan and colleagues in humans with primary aldosteronism (Torresan et al., 2024).

**FIGURE 4:**
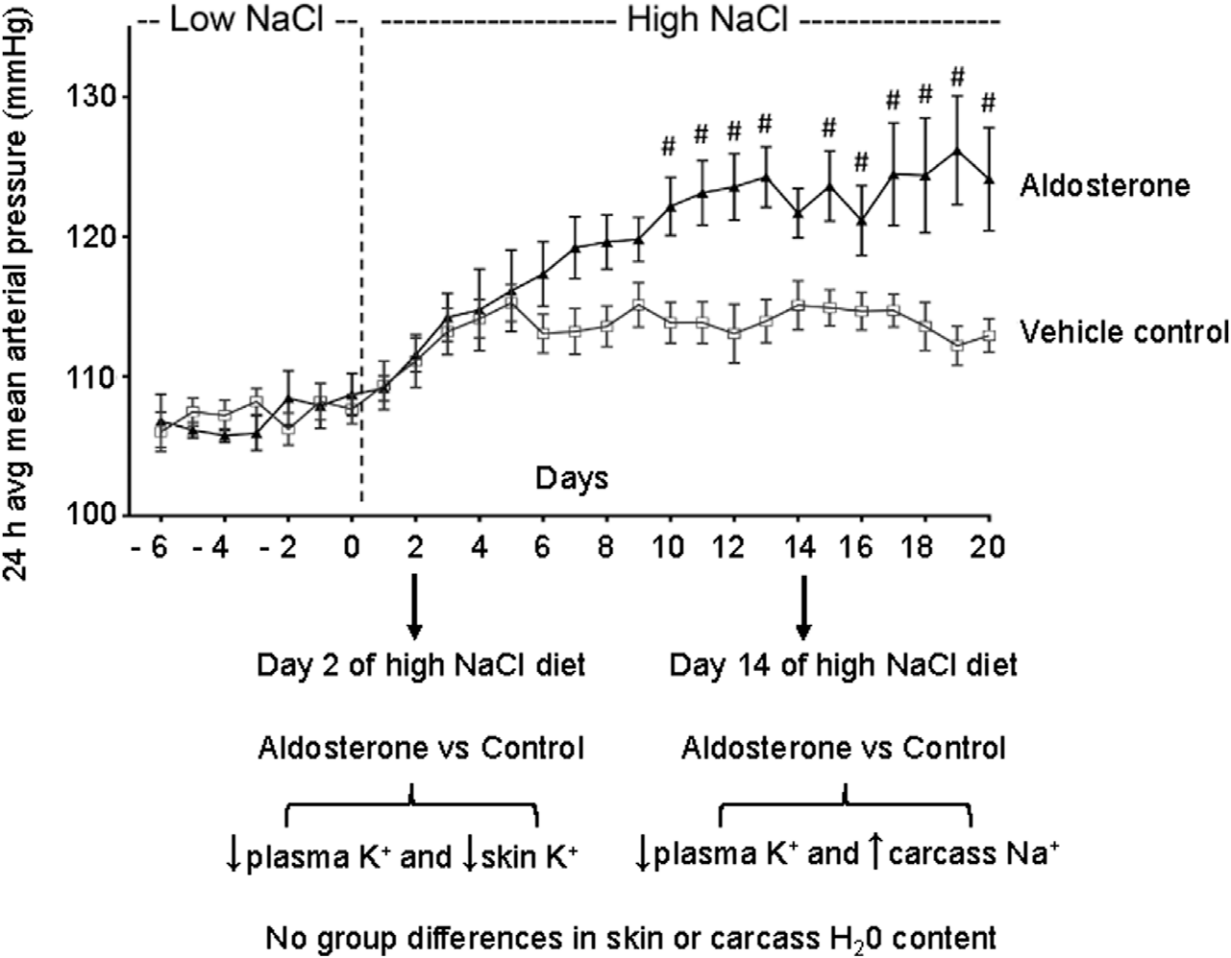
Relationship between time of sampling for tissue and plasma electrolytes and blood pressure. The figure shows the daily 24-h averages of mean arterial pressures (mean ± SEM) previously reported during administration of the low-NaCl diet (0.26% NaCl) and the high-NaCl diet (4% NaCl) in control rats infused with vehicle and in rats infused with aldosterone (Kurtz et al., 2023). After 2 days of salt loading in the present study, aldosterone-treated rats showed reduced plasma and skin potassium concentrations. After 14 days of salt loading, aldosterone-treated rats showed lower plasma potassium concentrations and higher carcass sodium concentrations compared with controls. The # symbol denotes P < 0.05 for differences in mean arterial pressure between the aldosterone group vs. the vehicle control group on individual experimental days in our previously reported study (Kurtz et al., 2023). Figure adapted from Kurtz et al., 2023.

Notably, in the present study, the observed hypokalemia and skin potassium depletion occurred before the reported time of onset of aldosterone-dependent salt sensitivity, which typically develops after 7–10 days of salt loading in this model (Figure 4) (Kurtz et al., 2023). This temporal relationship suggests that disturbances in tissue potassium may contribute to the initiation of salt-induced hypertension in hyperaldosteronism. It is possible that potassium depletion of the vasculature in skin and in other organs might be involved in promoting the increases in vascular resistance that initiate salt sensitivity and salt-dependent hypertension in hyperaldosteronism (Büssemaker et al., 2010; Kurtz et al., 2023). Potassium depletion may increase vascular resistance and promote salt sensitivity through multiple mechanisms such as reducing nitric oxide activity and increasing sympathetic nervous system activity (Goto et al., 1981; Oberleithner et al., 2009; Houston, 2011; Kurtz et al., 2021). Although hypokalemia is estimated to be present in only 9%–37% of cases of primary aldosteronism (Gruber and Beuschlein, 2020), it is possible for reductions in tissue potassium to occur in the absence of hypokalemia (Nørgaard and Kjeldsen, 1991; Bilbrey et al., 1973).

### 4.2 Skin sodium

Our study did not detect changes in skin sodium concentrations concurrent with the observed alterations in potassium. However, this does not preclude the possibility that sodium changes in other tissues could be involved in mediating increased vascular resistance and blood pressure in hyperaldosteronism. On day 14, although skin and plasma sodium levels were unchanged, carcass sodium concentrations were significantly increased in aldosterone-treated rats. Similar findings have been reported in deoxycorticosterone acetate-salt treated rats, where prolonged mineralocorticoid-salt treatment leads to sodium accumulation and potassium loss (with the potassium losses occurring primarily from skin) (Titze et al., 2005). However, given that these measurements were taken during the established phase of hypertension, the changes might reflect consequences of the disease process or mechanisms involved in its maintenance rather than its initiation.

### 4.3 Gene expression profiling in the skin

Gene set enrichment analysis after 2 days of salt loading revealed downregulation of biological processes related to anion homeostasis and hyperosmotic responses in aldosterone-treated animals compared with vehicle-treated controls. These changes may reflect early disturbances in anion and osmotic balance that contribute to the initiation of aldosterone-dependent salt-sensitive hypertension or may be secondary to early potassium depletion or both. After 14 days, the upregulation of genes influencing biological processes involved in muscle function and cellular calcium handling are of particular interest because of their potential relevance to vascular contractility and vascular resistance. Although a variety of the biological processes are associated with skeletal muscle function, they can also be involved in regulating vascular smooth muscle contraction.

Example of specific genes involved in smooth muscle function or calcium transport that were found to be significantly upregulated in aldosterone-treated animals include:

• *Cav3* (caveolin 3), which can influence the phenotypic switch between contractile and synthetic phenotypes of vascular smooth muscle cells (Gutierrez-Pajares et al., 2015).
• *Ryr1* (ryanodine receptor 1), which contributes to calcium signaling essential for muscle contraction and relaxation (Guerrero-Hernández et al., 2002).
• *Bves* (blood vessel epicardial substance), which plays a significant role in maintaining the contractile phenotype of VSMCs (Liu et al., 2022).
• *Ppp3cc* (protein phosphatase 3 catalytic subunit gamma), the gene encoding the gamma isoform of calcineurin, a calcium-dependent, calmodulin-stimulated protein phosphatase. In smooth muscle cells, calcineurin, through its catalytic subunits (including gamma), dephosphorylates target proteins, influencing muscle contraction and relaxation. This regulation is important for maintaining vascular tone and responding to physiological stimuli (Panchatcharam et al., 2013).
• *Smtnl1* (smoothelin-like 1), which is involved in mediating vascular smooth muscle contractile responses to intraluminal pressure (Turner et al., 2019).
• *Atp1a2*, (ATPase, Na^+^/K^+^ transporting, alpha 2 polypeptide) which regulates cellular calcium through its effects on the NCX (sodium-calcium exchanger) (Zhang et al., 2005).
• *Stim1* (stromal interaction molecule 1), which has an essential role in Ca (2+) homeostasis and in controlling function, growth, and development of smooth muscle cells (Kassan et al., 2016; Mancarella et al., 2013).

The upregulation of these genes aligns with evidence that aldosterone amplifies salt-induced increases in blood pressure by amplifying the increases in systemic vascular resistance that mediate initiation and maintenance of the salt-dependent hypertension (Kurtz et al., 2023).

### 4.4 Limitations

While we observed significant group differences in plasma and skin potassium after 2 days of salt loading, we did not detect clear group differences in plasma and skin sodium concentrations at either 2 days or 14 days of salt loading. This may be due to the relatively small sample sizes and limited statistical power of these studies. Future studies with larger sample sizes are needed to more reliably estimate potential differences in sodium concentrations. Additionally, our study focused on only two time points. More frequent electrolyte measurements together with telemetry studies would help clarify the detailed temporal relationship between electrolyte changes and blood pressure alterations.

The study focused on aldosterone-dependent effects on electrolyte metabolism in rats fed a high salt diet. Therefore, we compared animals on a high salt diet given aldosterone to animals on a high salt diet not given aldosterone. In future studies, it would be of interest to study control groups maintained on a low-salt diet throughout the study (with or without aldosterone treatment). External validation of our findings in humans with primary aldosteronism will be required to establish the clinical translational relevance of changes in electrolyte metabolism to the pathogenesis of aldosterone-dependent salt sensitivity. In addition, further work is needed to explore mechanistic links between electrolyte disturbances and the development of aldosterone-dependent salt sensitivity.

## 5 Conclusion

The present findings provide insights into the early electrolyte disturbances that occur in response to the combination of hyperaldosteronism and a high salt diet in a rat model of primary aldosteronism. As early as 2 days after initiating a high-salt diet, aldosterone-treated rats compared with vehicle treated controls, exhibited significant potassium depletion in skin and plasma, without concurrent sodium accumulation. Because substantial potassium depletion becomes evident before the point when blood pressure elevations are known to occur in this model, the findings suggest that potassium depletion may play a critical role in the initiation of aldosterone-dependent salt sensitivity and hypertension. These observations are consistent with recent reports that in humans, surgical cure of unilateral primary aldosteronism is associated with significant increases in skin and plasma potassium concentrations without changes in sodium levels (Torresan et al., 2024). Gene set enrichment analysis suggests that disturbances in ion transport and regulation of contractile function might be involved in mediating the aldosterone-dependent salt sensitivity and hypertension. The results warrant further detailed time-course studies across various tissues to elucidate the mechanistic and temporal relationships between electrolyte levels and the abnormalities in vascular resistance that cause salt sensitivity and hypertension in hyperaldosteronism.

## Data Availability

The gene expression data are available in ArrayExpress database https://www.ebi.ac.uk/biostudies/arrayexpress website (accession number E-MTAB-14749).
