# Supplementary material for: Early reduction of skin potassium without sodium accumulation in the pathogenesis of salt sensitivity in primary aldosteronism
Source: Front Pharmacol. 2025 Apr 29;16:1575972. doi: 10.3389/fphar.2025.1575972 (PMC12069341; doi:10.3389/fphar.2025.1575972)

Salt loading protocol in vehicle control group

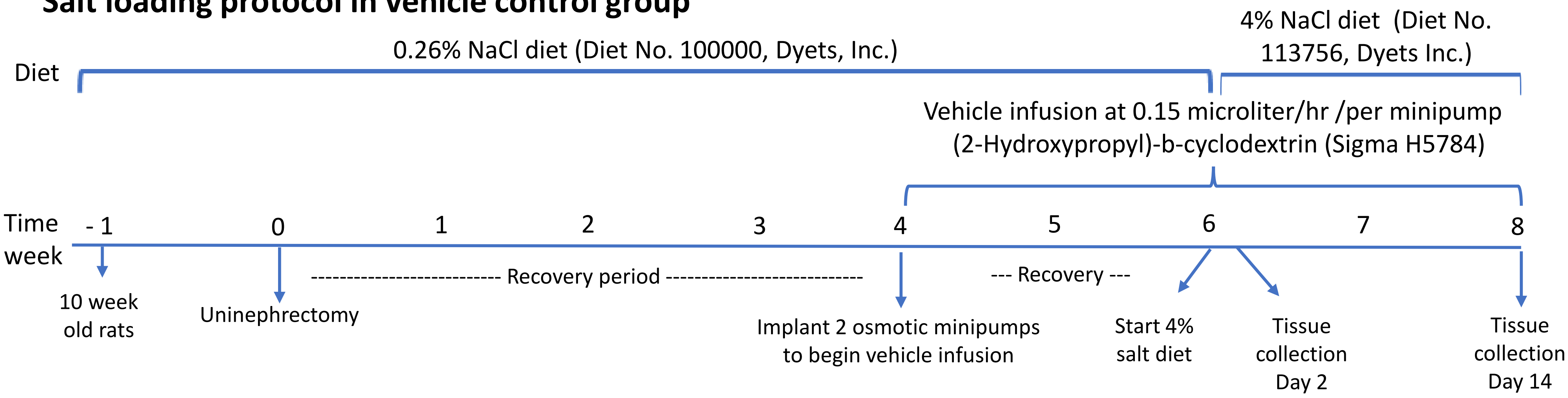

Salt loading protocol in aldosterone treatment group

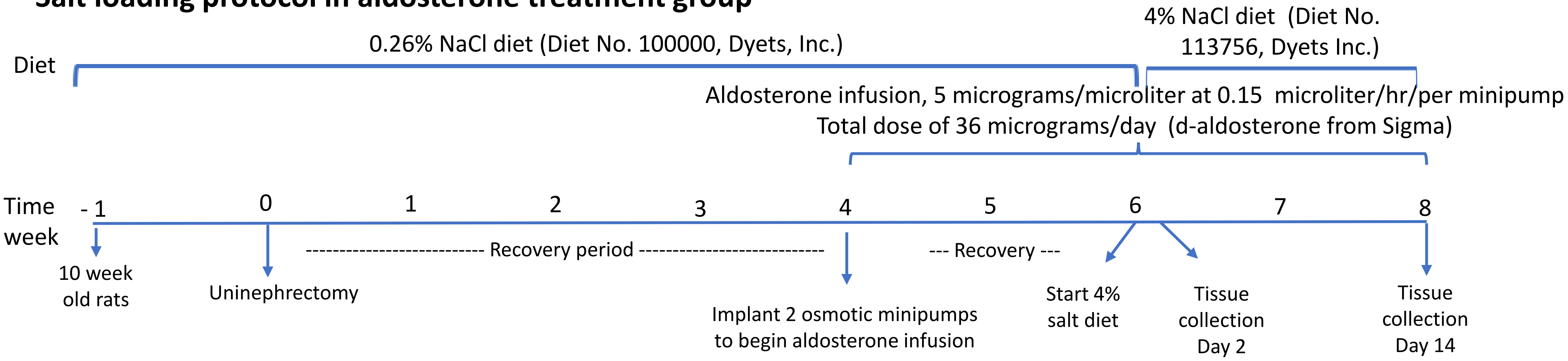

Supplement: Supplementary file 1 [file DataSheet1.pdf]
